# Human PIF1 helicase supports DNA replication and cell growth under oncogenic-stress

**DOI:** 10.18632/oncotarget.2501

**Published:** 2014-11-05

**Authors:** Mary E. Gagou, Anil Ganesh, Geraldine Phear, Darren Robinson, Eva Petermann, Angela Cox, Mark Meuth

**Affiliations:** ^1^ Academic Unit of Molecular Oncology, Department of Oncology, School of Medicine and Biomedical Sciences, University of Sheffield, Sheffield, UK; ^2^ Light Microscopy Facility, Department of Biomedical Science, University of Sheffield, Firth Court, Sheffield, UK; ^3^ School of Cancer Sciences, University of Birmingham, Edgbaston, Birmingham, UK

**Keywords:** helicases, replication stress, oncogenes, human cells

## Abstract

Unwinding duplex DNA is a critical processing step during replication, repair and transcription. Pif1 are highly conserved non-processive 5′->3′ DNA helicases with well-established roles in maintenance of yeast genome stability. However, the function of the sole member of Pif1 family in humans remains unclear. Human PIF1 is essential for tumour cell viability, particularly during replication stress, but is dispensable in non-cancerous cells and *Pif1* deficient mice. Here we report that suppression of PIF1 function slows replication fork rates and increases arrested forks during normal cycling conditions. Importantly, PIF1-dependent replication impediments impair S-phase progression and reduce proliferation rates of RAS oncogene-transformed fibroblasts, where replication fork slowing is exacerbated, but not parental, non-cancerous cells. Disrupted fork movement upon PIF1-depletion does not enhance double-stranded break formation or DNA damage responses but affects resumption of DNA synthesis after prolonged replication inhibitor exposure, accompanied by diminished new origin firing and mainly S-phase entry. Taken together, we characterised a functional role for human PIF1 in DNA replication that becomes important for cell growth under oncogenic stress. Given that oncogenes induce high levels of replication stress during the early stages of tumorigenesis, this function of PIF1 could become critical during cancer development.

## INTRODUCTION

Human cells encode a number of helicases that catalyse the unwinding of DNA in a directionally specific manner. Several of these are essential for cell growth and survival whereas loss or mutation of others have been causally related to genetic disorders with cancer predisposition [1] including Blooms [2], Werners [3], Rothmund-Thomson [4] and Fanconi's Anemia [5] syndromes. The highly conserved Pif1 proteins protect genome stability, by regulating telomere homeostasis [6], Okazaki fragment maturation [7], G-quadruplex DNA (G4-DNA) resolution [8, 9], replication through DNA-protein barriers and highly transcribed genes [10, 11] and, replication induced by DNA double-strand breaks (DBS) [12, 13]. However, it is unclear whether the above functions have been conserved in all proteins of the Pif1 family. A pronounced example is the distinct roles of the ScPif1 and ScRrm3 proteins in DNA replication of the yeast *Saccharomyces cerevisiae* genome [14–16]. This also reflects the fact that although Pif1 proteins perform multiple tasks in DNA metabolism, not all of them are essential for cell viability [14, 17, 18].

The human genome encodes a single PIF1 gene. Its expression, via alternative splicing, gives rise to two transcriptional isoforms, translation of which results in two protein isoforms with different subcellular localisation [19]. The major transcript, hPif1a, encodes a nuclear polypeptide of approximately 70kDa [19–21]. The second transcript, hPif1b, encodes a polypeptide of approximately 75kDa, highly enriched in mitochondria [19]. A second mitochondrial polypeptide, approximately 45kDa, can be generated from the hPif1a, by downstream Alternative Translation Initiation [22]. It is worth mentioning that the presence, function and physiological significance of the two mitochondrial PIF1 isoforms remain unclear since they have not been conserved in mouse [17]. It is possible that their expression is limited and/or is restricted to specific human tissues since they have been identified only in the two above studies, respectively. For the purpose of clarity, we will further refer to 70kDa nuclear isoform, as the human PIF1 protein.

Concerning its biochemical properties and biological significance, human PIF1 shares common *in vitro* substrates with the yeast Pif1 proteins, having specificity for telomeric DNA [21], synthetic stalled DNA replication fork-like structures [23, 24], but mainly for G4-DNA [25]. We have shown [26] that siRNA-mediated PIF1-depletion results in a combination of apoptosis, reduced survival, hypersensitivity to therapeutic DNA replication inhibitors and defective cell cycle progression in several cancer cell lines independent of p53 status. Importantly, non-cancerous cells did not show a similar response.

We reasoned that PIF1 could affect DNA replication in a way that becomes particularly critical only during replication stress experienced by cancer cells. Given that oncogene expression induces replication stress during early stages of tumorigenesis [27, 28] we aimed to investigate whether specific oncogene transformations of non-cancerous cells could trigger PIF1-dependent mechanism(s) of DNA replication progression and recovery, ensuring cell growth and proliferation. We found that PIF1 functions to maintain undisrupted DNA replication fork progression during normal cycling conditions and to support resumption of DNA synthesis after prolonged S-phase arrest. The dependence of DNA replication on PIF1 function increases under oncogene overexpression.

## RESULTS

### PIF1-depletion impairs genome-wide DNA replication progression during unperturbed conditions

**Figure 1:**
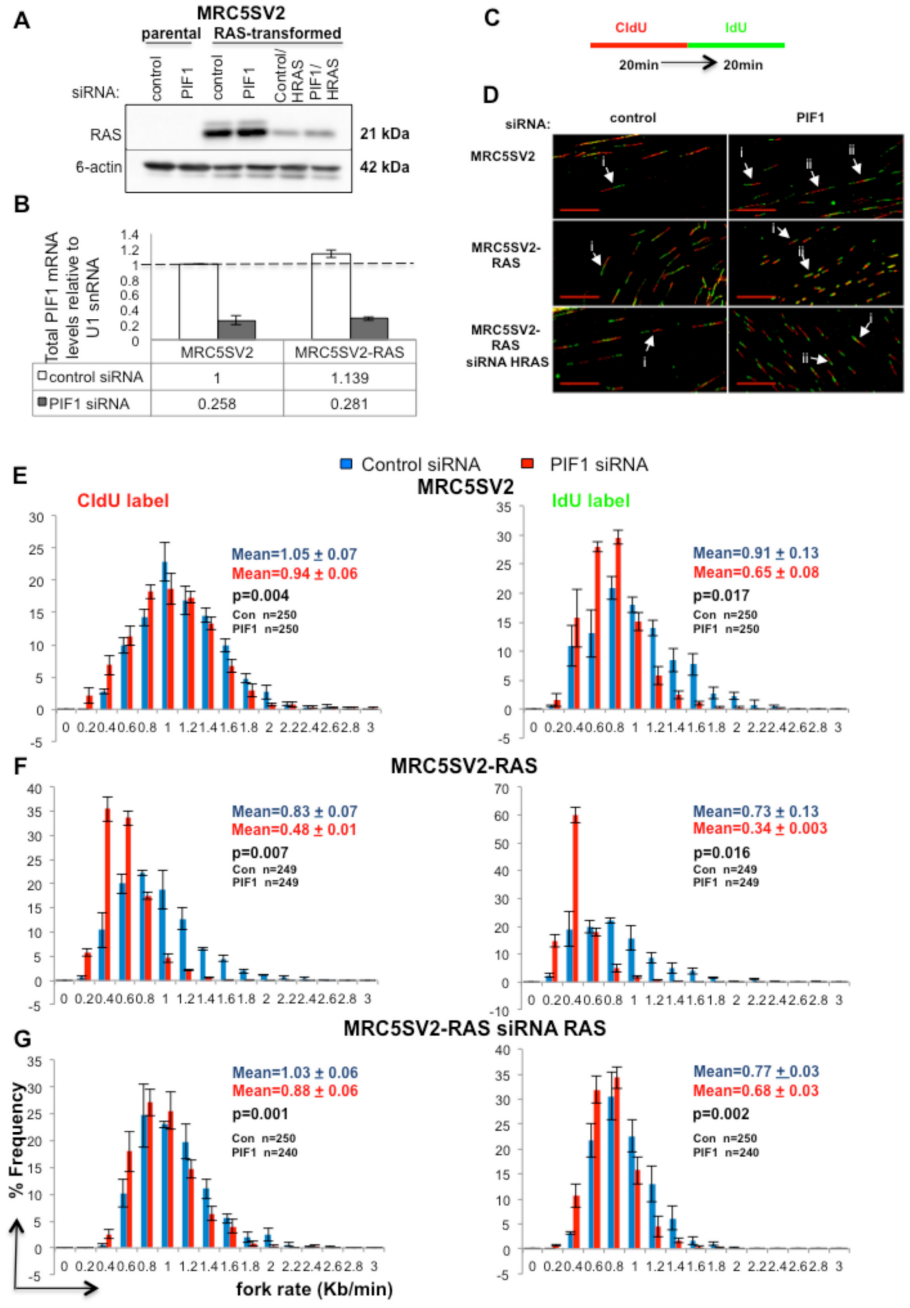
PIF1 depletion slows DNA replication fork rates under normal cycling conditions **(A)** Western blot analysis of RAS expression in parental and H-RAS^G12V^-transformed (clone 2) MRC5SV2 cells after indicated siRNA treatments. ϐ-actin levels served as loading controls, **(B)** Quantitative RT-PCR analysis of total PIF1mRNA, **(C)** Labelling protocol of parental and MRC5SV2-RAS cells after indicated treatment, **(D)** Representative images (scale bars=20μm) of replication tracks. White arrows marked with (**i**) and (**ii**) indicate single forks containing tracks from the 1^st^ and 2^nd^ pulses (red-green) and, bidirectional forks (green-red-green) generated in the 1^st^ pulse, respectively, **(E-G)** Distribution of fork rates during CIdU and IdU pulses is shown in left and right panels, respectively. Fork rates were calculated as the length of the labelled track divided by the time of the pulse. Data bars present the mean of three independent experiments and error bars represent SD in B and SEM in panels E-G. In fork rate distributions, the total mean and SD of the three repeats are also shown, as well as *p* values of Student's *t* test. The exact numbers (n) for each experiment are listed as well.

For our experimental system, we over-expressed the mutated form of the proto-oncogene RAS, H-RAS^G12V^, commonly found in human tumours [29], in immortalized MRC5SV2 human fibroblasts. RAS stable protein expression was confirmed by Western blot analysis (Fig. 1A). Suppression of PIF1 function was performed by siRNA-mediated depletions. Due to the limited levels of the endogenous PIF1 protein that make its detection by immunoblotting difficult, as we and others have shown [20, 26], the efficiency of the siRNA treatments was determined by quantitative PCR (qPCR) (Fig. 1B and Supplementary Information Text and Fig. S1A-E).

To test our hypothesis, we applied genome-wide DNA-fibre analysis [30]. In a double-label assay, parental and RAS-oncogene-transformed fibroblasts, after transfection with control or PIF1 siRNAs, were pulse-labelled with the thymidine analogues 5-Chloro-2′-deoxyuridine (CIdU) and 5-Iodo-2′-deoxyuridine (IdU) (Fig. 1C), and the length of labelled tracks on DNA spreads was measured by immunofluorescence analysis. Fork rates were assessed from ongoing replication structures (Fig. 1D). During an unperturbed cell cycle, replication elongation rates were slower in parental fibroblasts, after PIF1-depletion: a significant shift of the entire distribution of fork rates was observed in PIF1 knockdowns relative to control, leftwards to slower rates (Fig. 1E). This was more pronounced during the second pulse, where the average fork speed of control siRNA treated cells was reduced from 0.91kb/min to 0.65kb/min (p=0.017) after PIF1 siRNA treatment.

Fork rate slowing upon PIF1-depletion was greatly exacerbated in RAS-transformed fibroblasts, where the average rate of replication fork progression diminished by about half, relative to control, during both pulses [%percentage reduction of fork rates: 42.17 (p=0.007) and 53.42 (p=0.016) in CIdU and IdU tracks, respectively, (Fig. 1F)]. This exacerbation was specific to RAS overexpression since simultaneous treatment of RAS-transformed fibroblasts with HRAS and control or PIF1 siRNAs restored fork rates to parental levels (Fig. 1G). In particular, the average fork rate of CIdU tracks in RAS-transformed cells co-treated with control and HRAS siRNAs was 1.03kb/min relative to 1.05kb/min (p=0.275) in control siRNA-treated parental cells, while co-treatment of RAS-transformed cells with PIF1 and HRAS siRNAs produced a rate of 0.88kb/min vs 0.94kb/min (p=0.175) in PIF1 siRNA-transfected parental cells. Similarly, measurements of IdU tracks showed that co-treatment with control and HRAS siRNAs in RAS-transformed cells leads to an average fork speed of 0.77kb/min relative to 0.91kb/min (p=0.216) in control siRNA parental cells, while co-treatment of RAS-transformed cells with PIF1 and HRAS siRNAs resulted to 0.68 vs 0.65 (p=0.487) in PIF1 siRNA parental cells. Additionally, similar results were obtained with a second clonal derivative of MRC5SV2 expressing RAS (clone 3), where the effects of PIF1 depletion on replication fork movement were studied after deconvolution of the siRNA pools used above (Supplementary Fig. S2A-C and S3A-D). In all the above assays, we observed a greater effect of siRNA treatments on fork rate slowing during the second pulse relative to the first one. A reported explanation [31] for this is that by measuring only double-labelled structures (red-green and, green-red-green), we exclude all the pausing events that can occur during the first pulse and lead to unilabelled tracks (only red). However, structures with stalled/termination events upon the second pulse are included because they were already labelled from the first pulse [31].

**Figure 2:**
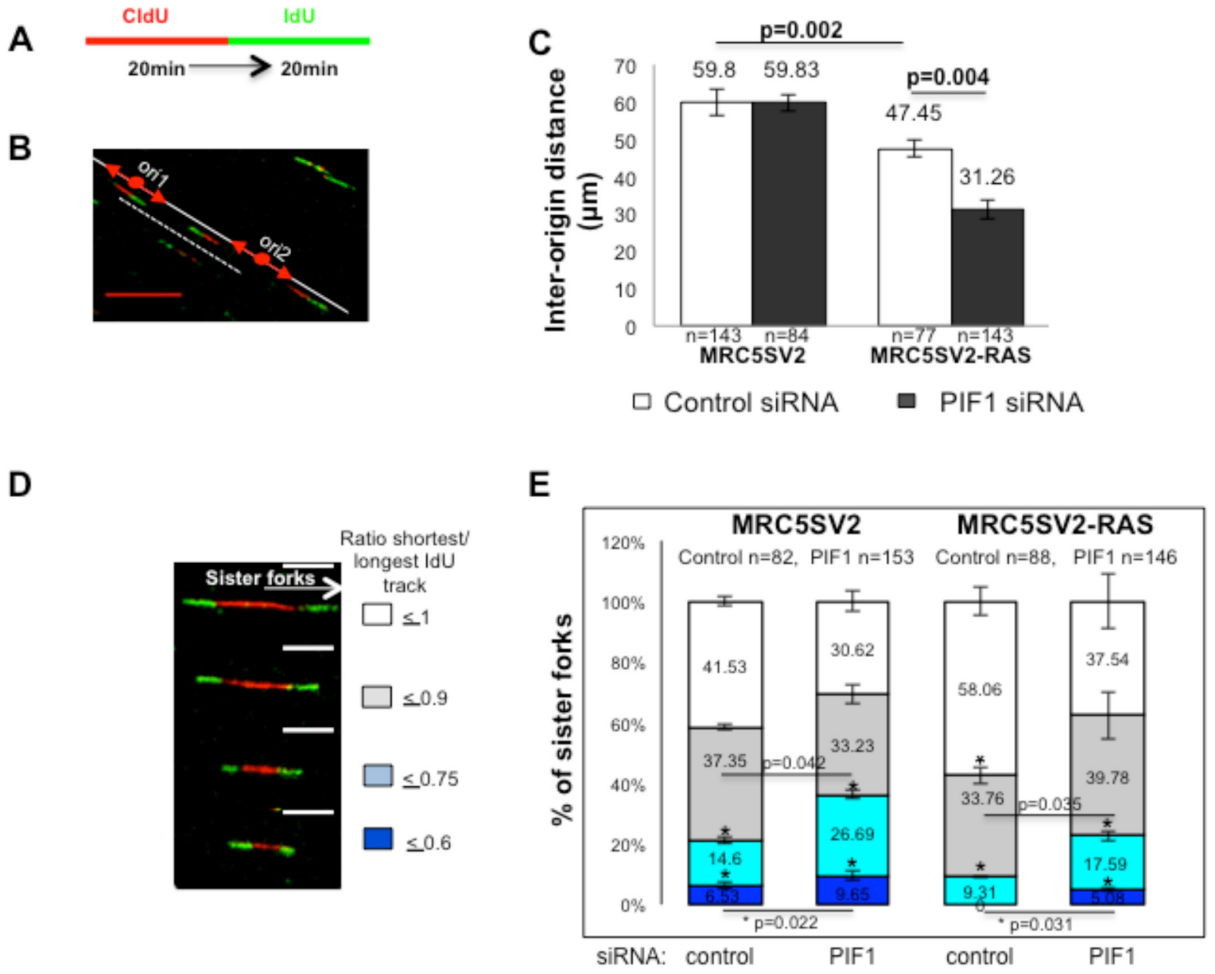
PIF1 depletion changes the frequencies of activation of new replication origins and fork stalling under conditions of oncogene overexpression **(A)** Labelling protocol of parental and MRC5SV2-RAS cells (clone 2) after indicated treatment, **(B)** Representative image (scale bars=10μm) of origin-to-origin distance (marked with a white dashed line) in MRC5SV2-RAS cells treated with PIF1 siRNAs, **(C)** Bar graphs with inter-origin distances, **(D)** Representative images (scale bars=5μm) and, **(E)** Bar graphs with shown percentages of symmetric and asymmetric progression of sister forks initiated from the same origin during the 1^st^ pulse. Data bars present the mean of three independent experiments and error bars represent SD. *p* values of Student's *t* test and the exact numbers (n) for each experiment are listed as well.

To examine whether slow fork speed was associated with further changes of DNA replication, in the above assays we measured inter-origin distances as an indication of new origin activation (Fig. 2A-C). We found that origin-to-origin distances were reduced in RAS transformed fibroblasts relative to parental cells (average distance: 47.45μm and 59.8μm (p=0.002) in MRC5SV2-RAS and MRC5SV2 cells, respectively), in accord to the previously reported aberrant replication initiation during oncogene overexpression [28]. Inter-origin distances were further reduced upon PIF1 depletion in RAS transformed fibroblasts, relative to control, (average distance: 31.26μm vs 47.45μm (p=0.004) in PIF1 or control siRNA MRC5SV2-RAS cells, respectively), implying that new origin firing was increased to compensate the slow fork rates in these cells. By contrast, inter-origin distances were unaffected after PIF1 depletion in parental cells (average distance: 59.83μm in both PIF1 or control siRNA treated MRC5SV2 cells), where a modest reduction of fork speed was observed.

In the above assays of parental and RAS-transformed cells, we also measured the symmetry of progression of sister forks initiated from the same origin (Fig. 2D, E). As previously defined [32], we considered symmetric progression of the two forks when the ratio of shorter to longer IdU track was > 0.75. Replication structures with poor symmetry (≤ 0.75) were increased significantly upon PIF1-depletion in parental and RAS-transformed fibroblasts indicating increased fork stalling. In particular, the fold increase of total structures with poor symmetry after PIF1 depletion was 1.72 and 2.43 in parental and RAS-transformed cells, respectively.

**Figure 3:**
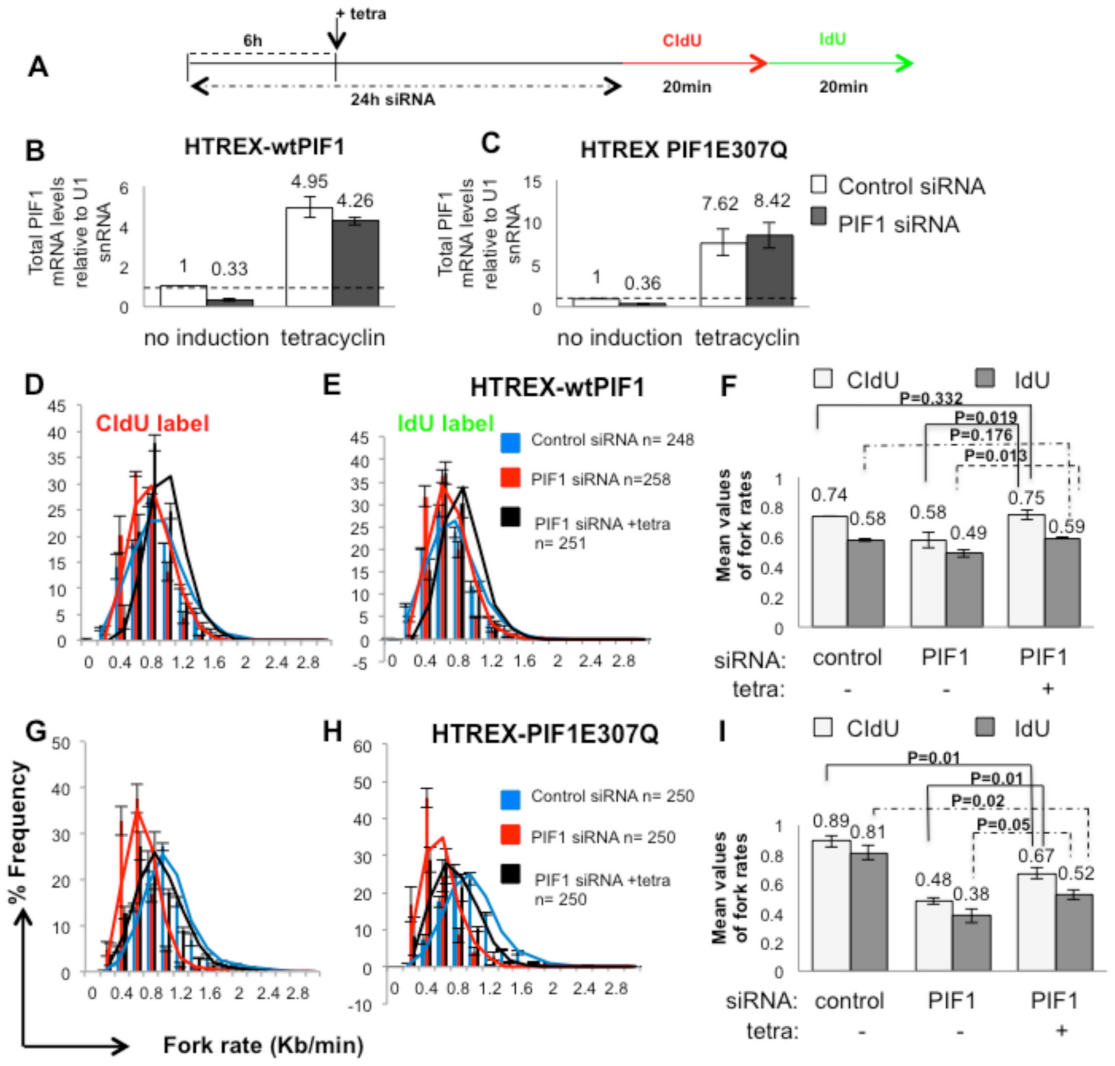
Decreased replication rates upon PIF1 depletion were restored in HCT116 cells by overexpression of wild type PIF1 protein while a helicase mutant failed to restore replication to wild type levels **(A)** Labelling protocol of HCT116 cell lines (HTREX-wtPIF1 and HTREX-PIF1E307Q) transfected with control or a siRNA duplex targeting a 3′ UTR region of PIF1 (PIF1 siRNA 2) for 6h before induction of expression of siRNA-resistant recombinant wtPIF1 or mutant PIF1E307Q proteins, respectively. Cells left to grow for another 18h before labelling with CIdU and IdU for the indicated time, **(B, C)** qRT-PCR analysis of total PIF1mRNA, relative to U1 snRNA, in HTREX-wtPIF1 and HTREX-PIF1E307Q cells, respectively. **(D-I)** Distributions and mean values of fork rates during both pulses in HTREX-wtPIF1**(D-F**) and HTREX-PIF1E307Q cell lines (**G-I**), respectively. Data bars are the mean of three independent experiments and (n) the exact number of replication structures measured in each repeat. Error bars represent SD in **B-C, F** and **I** Panels and SEM in **D-E** and **G-H** Panels. *P* values of Student's *t* test are also presented.

Slower fork rates were also detected in HCT116 tumour cells (with an activating K-RAS^G13D^ mutation) following PIF1-depletion [average fork rate: 0.58kb/min vs 0.74kb/min (p=0.046) for CIdU tracks and 0.49kb/min vs 0.58kb/min (p=0.041) for IdU tracks in PIF1 and control siRNA treated HCT116 cells (Fig. 3A-I)]. Replication fork progression was restored by induced overexpression of a siRNA-resistant recombinant of the wild type PIF1, confirming specificity of siRNA treatments (Fig. 3A, B and D-F). In particular, in both un-induced and tetracyclin-induced cells following treatment with control or PIF1 siRNAs, respectively, the average fork rate was 0.7kb/min (p=0.332) in CIdU tracks and 0.6kb/min (p=0.176) in IdU tracks. In contrast, HCT116 cells carrying an inducible PIF1 E307Q helicase mutant (that abolished helicase activity while retaining DNA binding ability [24], failed to correct fork rates to wild type levels after induction [average fork rate: 0.67kb/min vs 0.89kb/min (p=0.01) of CIdU tracks and 0.52kb/min vs 0.81kb/min (p=0.02) of IdU tracks in induced cells treated with PIF1 siRNA and un-induced cells treated with control siRNA, respectively (Fig. 3A, C and G-I)]. However, fork rates were weakly increased in PIF1-depleted cells conditionally expressing the mutant protein [average fork rate: 0.67kb/min vs 0.48kb/min (p=0.01) of CIdU tracks and 0.52kb/min vs 0.38kb/min (p=0.05) of IdU tracks in PIF1 siRNA treated cells after induction or not of the mutant PIF1, respectively. This could potentially be due to the annealing activity of the PIF1 helicase that is not affected by the E307Q mutation [24].

Taken together, our data show that, during an unperturbed cell cycle, PIF1-depletion affects DNA replication by slowing fork rates, especially in oncogene-expressing cells, and by increasing fork stalling. Importantly, the function of PIF1 in maintenance of replication fork progression requires, its helicase activity.

### PIF1 depletion increases fork stalling during ligand-induced stabilization of G4-structures

**Figure 4:**
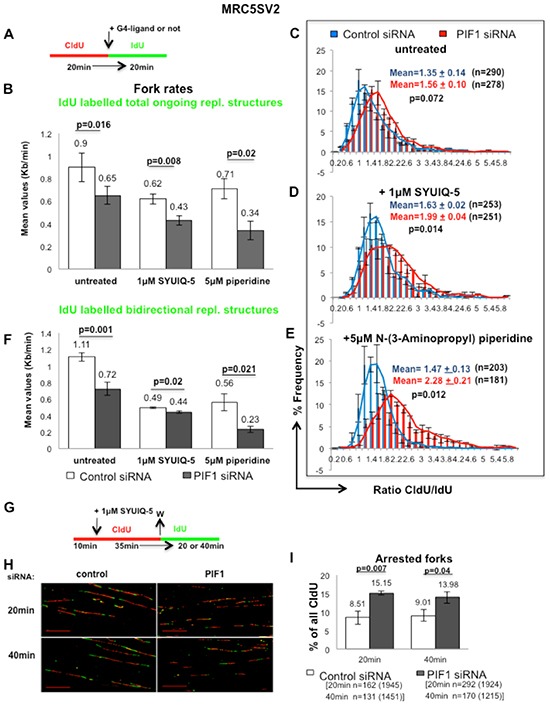
PIF1 protects forks from stalling during ligand-induced stabilization of G4-structures **(A)** Labelling protocol of MRC5SV2 cells treated with control or PIF1 siRNAs and sequentially labelled with CIdU and IdU in the presence or not of 1μM SYUIQ-5 or 5μM N-(3-Aminopropyl) piperidine, **(B)** Bar graphs show mean values of fork rates during IdU pulse, measured as in Figure 3, **(C-E)** Distribution plots of CIdU/IdU ratio, **(F)** Bar graphs show mean values of fork rates during IdU pulse, measured only in bidirectional replication structures (green-red-green), **(G)** Labelling protocol and, **(H)** Representative images (scale bars=20μm) of replication structures of MRC5SV2 cells treated with control or PIF1 siRNAs and pulsed with CIdU in the presence of 1μM SYUIQ-5. After ligand removal, cells were pulsed with IdU for the indicated times, **(I)** Bar graphs with mean values of arrested forks/termination events, assessed as percentage of forks that progressed only during the 1^st^ pulse (red) relevant to all CIdU tracks. Data bars present means of three independent experiments and error bars represent SD in **B, F**, **I** and SEM in **C-E**. In CIdU/IdU ratio distributions, the total mean and SD of the three repeats are also shown, as well as *p* values of Student's *t* test. The exact numbers (n) of each experiment are listed as well. In panels **B** and **F** the exact numbers are the same with these in panels **C-E**. In panel **I**, n is the number of the total arrested forks pooled from three independent experiments and in parenthesis the number of the total CIdU-labelled replication structures (red-green, green-red-green and only red) is presented as well.

We next investigated whether impairment of fork movement upon PIF1 depletion is affected by ligand-induced stabilization of G4-structures, given the *in vitro* preference of human PIF1 for unwinding these structures [25] and, the supporting role of ScPif1 on DNA replication through G4-motifs [33]. Modifying our previous fibre-assay, non-cancerous fibroblasts were sequentially pulsed with CIdU and IdU for equal periods of time, but during the second pulse a G4-ligand was added (Fig. 4A). We have used two different commercially available ligands, the quindoline derivative SYUIQ-05 and the berberine derivative N-(3-Aminopropyl) piperidine. Both small molecules induce formation and stabilisation of G4-structures at telomeres [34, 35] as well as at non-telomeric regions [36, 37]. Replication slowing upon ligand treatments resulted in shortening of the IdU track relative to CIdU one. In particular, in the presence of 1μM SYUIQ-05 or 5μM N-(3-Aminopropyl) piperidine and following PIF1-depletion, a significant reduction in fork rates of ongoing replication structures was observed, relative to control and untreated cells (Fig. 4B), leading to a significant increase in the ratio of CIdU/IdU track lengths, respectively (Fig. 4C-E). We found that the percentage reduction of the fork speed of IdU tracks after PIF1 depletion, relative to control, was 27.78% (p=0.016) in untreated MRC5SV2 cells and further increased to 30.64% (p=0.008) and 52.11% (p=0.02) in the presence of 1μM SYUIQ-05 5μM or N-(3-Aminopropyl) piperidine, respectively. Importantly, reduced fork rates under the above conditions were also observed in ongoing replication structures initiated bi-directionally during the first pulse-label (Fig. 4F). This excludes the possibility that the above effect is due to the function of G4-ligands only at telomeres, given that DNA replication origins at telomeres are unidirectional [38]. Following PIF1 depletion, DNA replication elongation rates were reduced 35.13% (p=0.001), relative to control, in untreated cells while in the presence of DNA ligands, the reduction was 10.20% (p=0.02) and 58.93% (p=0.021) in SYUIQ-05 5μM or N-(3-Aminopropyl) piperidine treated cells, respectively. The limited effect of SYUIQ-05 on fork rates of non-telomeric regions could be explained by the preference of this ligand for binding on a particular G4-region [36]. However, SYUIQ-05 treatment significantly increased the frequency of arrested forks in PIF1-depleted cells relative to control (Fig. 4G-I). Arrested forks were considered only as replication structures which progressed during the first pulse and failed to progress during the second. We found that upon PIF1 depletion, arrested forks increased 78.02% (p=0.007) and 55.16% (p=0.04), relative to control, 20min or 40min after removal of SYUIQ-05 ligand, respectively. Taken together, our data suggest that PIF1 protects replication forks from stalling during ligand-induced stabilization of G4-structures.

### Depletion of PIF1 differentially affects cell growth and survival of parental and RAS-oncogene-transformed cells

**Figure 5:**
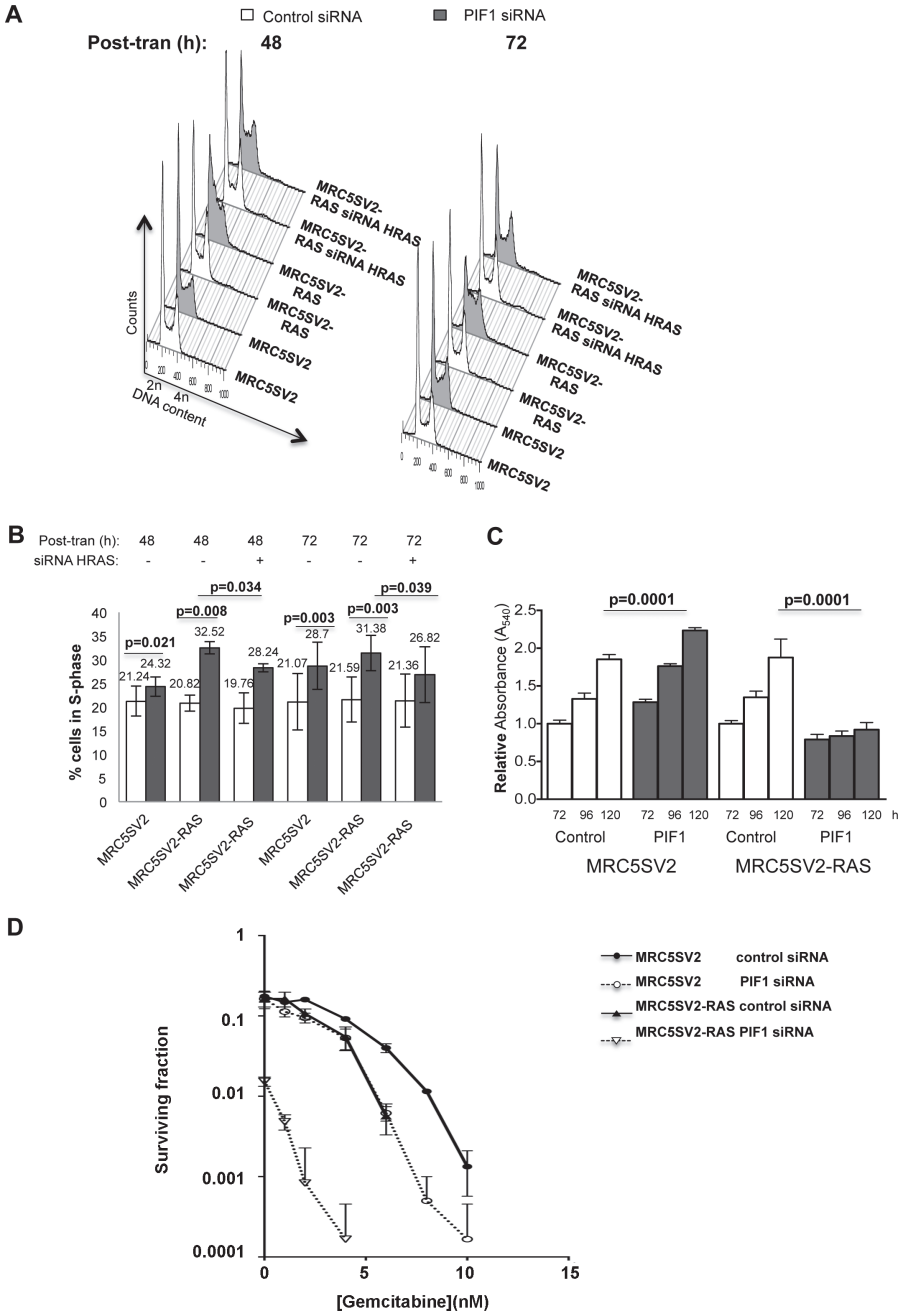
Differential effects of PIF1 depletion on growth and survival of parental and MRC5SV2-RAS cells (clone 2) Cells, after indicated siRNAs treatments, were left to grow for 48 or 72h before harvest for analysis of DNA content by flow cytometry. **(A)** Representative cell cycle profiles and, **(B)** Bar graphs showing the S-phase cell cycle distributions determined from these profiles, **(C)** Cell proliferation, determined by MTT assays, in parental and RAS-transformed fibroblasts transfected with control or PIF1 siRNAs. Indicated times correspond to post-transfection hours. Results represent means of at least three independent experiments with standard deviations indicated by error bars as well as *p* values of Student's *t* test, **(D)** Cells treated with indicated siRNAs were plated in medium supplied with gemcitabine at increasing concentrations and left to grow. Colonies of surviving cells were scored and are presented as surviving curves.

We next examined how the above defects in DNA replication progression impacted overall cellular growth, proliferation and survival upon PIF1-depletion. Consistent with the effects of PIF1-depletion on replication fork progression, FACS-based analysis of DNA content in parental fibroblasts, showed a slight but significant increase in the percentage of cells in S-phase after PIF1-depletion [24.32% vs 21.24% (p=0.021) after 48h and 28.7% vs 21.07% (p=0.003) after 72h transfection with PIF1 or control siRNAs, respectively, (Fig. 5A, B and Supplementary Information Table 1)]. This increase in S-phase cells was much more pronounced in RAS-transformed fibroblasts, where accumulation of cells in S-phase (particularly in early S-phase) was evident (relative to control). In particular, the percentage of cells in S-phase was 32.52% vs 20.82% (p=0.008) after 48h and 31.38% vs 21.59% (p=0.003) after 72h transfection with PIF1 or control siRNAs, respectively. Moreover, co-treatments of RAS-transformed fibroblasts with HRAS and PIF1 siRNAs reduced S-phase populations [%S-phase cells: 28.24 vs 32.52 (p=0.034) after 48h and 26.82 vs 31.38 (p=0.039) after 72h co-transfection with PIF1 and HRAS siRNAs or single transfection with PIF1 siRNAs, respectively]. The above results were also confirmed with measurements of BrdU incorporation in asynchronous cultures of parental and two different clones of RAS transformed fibroblasts treated with two different PIF1 siRNAs (Supplementary Information Fig. S4).

Furthermore, in MTT proliferation assays, metabolic activity nearly doubled over the time course in parental cells treated with control or PIF1 siRNAs and in RAS-transformed fibroblasts treated with the control siRNA. In contrast there was very little increase in activity upon PIF1-depletion of RAS-transformed fibroblasts (Fig. 5C). Moreover, there was a 10-fold reduction of colony formation by PIF1-depleted RAS-transformed fibroblasts relative to control or parental fibroblasts (Fig. 5D). These frequencies were further suppressed (106- to 180-fold) in PIF1-depleted RAS-transformed fibroblasts relative to control or parental cells after gemcitabine treatment (Fig. 5D).

Taken together, our data show that, during an unperturbed cell cycle, PIF1-depletion has much greater impact on the growth, proliferation and survival of RAS-transformed cells than on parental fibroblasts.

### DNA damage response and integrity of replication forks upon PIF1 depletion

**Figure 6:**
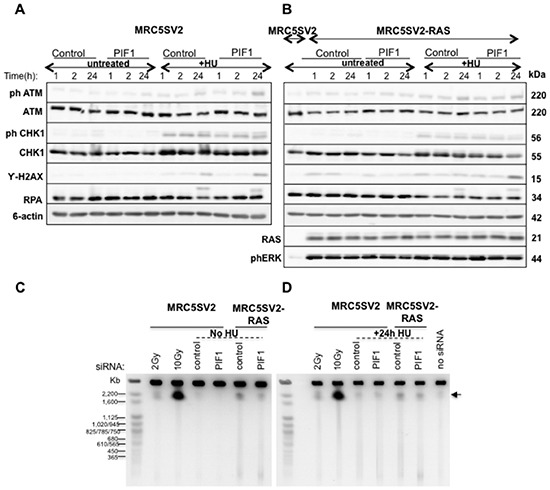
Increased fork stalling after PIF1 depletion is not associated with activation of DNA damage responses, or increased DSB formation **(A-B)** Immunoblot analysis of phospho-ATM, ATM, phospho-CHK1, CHK1, RPA2 and γ-H2AX in total protein extracts and, **(C-D)** DNA Double-Strand Break (DSB) formation analysis, by Pulse-field Gel Electrophoresis, in parental and MRC5SV2-RAS cells (clone 2) transfected with control or PIF1 siRNAs for 48h and treated or not with 2mM HU for indicated times. ϐ-actin levels served as loading controls. Parental cells with no siRNA treatment as well as cells irradiated with 2Gy or 10Gy were used as negative and positive controls for DSB formation, respectively. DSBs are indicated with an arrow.

We next questioned whether increased fork stalling provokes DNA damage response upon PIF1-depletion. Intriguingly, immunoblotting analyses showed that in normal cycling conditions PIF1-depletion does not trigger CHK1 activation, phosphorylation of histone H2AX, or hyperphosphorylation of RPA2 in either parental or RAS-transformed fibroblasts, while ATM phosphorylation was at control levels (Fig. 6A, B). Moreover, upon disruption of DNA replication with Hydroxyurea (HU) treatment, a weak increase of the levels of phosphorylated H2AX and ATM was observed in PIF1-depleted cells relative to control while CHK1 activation and RPA2 hyperphosphorylation were largely unaffected. In accord with the absence of significant DNA damage response after PIF1- depletion, pulse-field gel electrophoresis analyses showed similar levels of DSBs in control and PIF1 siRNA treated cells in both unperturbed growth conditions or after 24h HU treatment (Fig. 6C, D). An increase in DSB formation was detected in RAS-transformed fibroblasts relative to parental cells, consistent with previous reports [39] of induction of replication stress upon RAS overexpression. However, this was not further affected by PIF1-depletion. The above data imply that there is no widespread collapse of replication forks or induction of DNA damage upon PIF1-depletion despite the effects of PIF1 on DNA replication.

### PIF1 promotes recovery of prolonged-disrupted DNA replication

**Figure 7:**
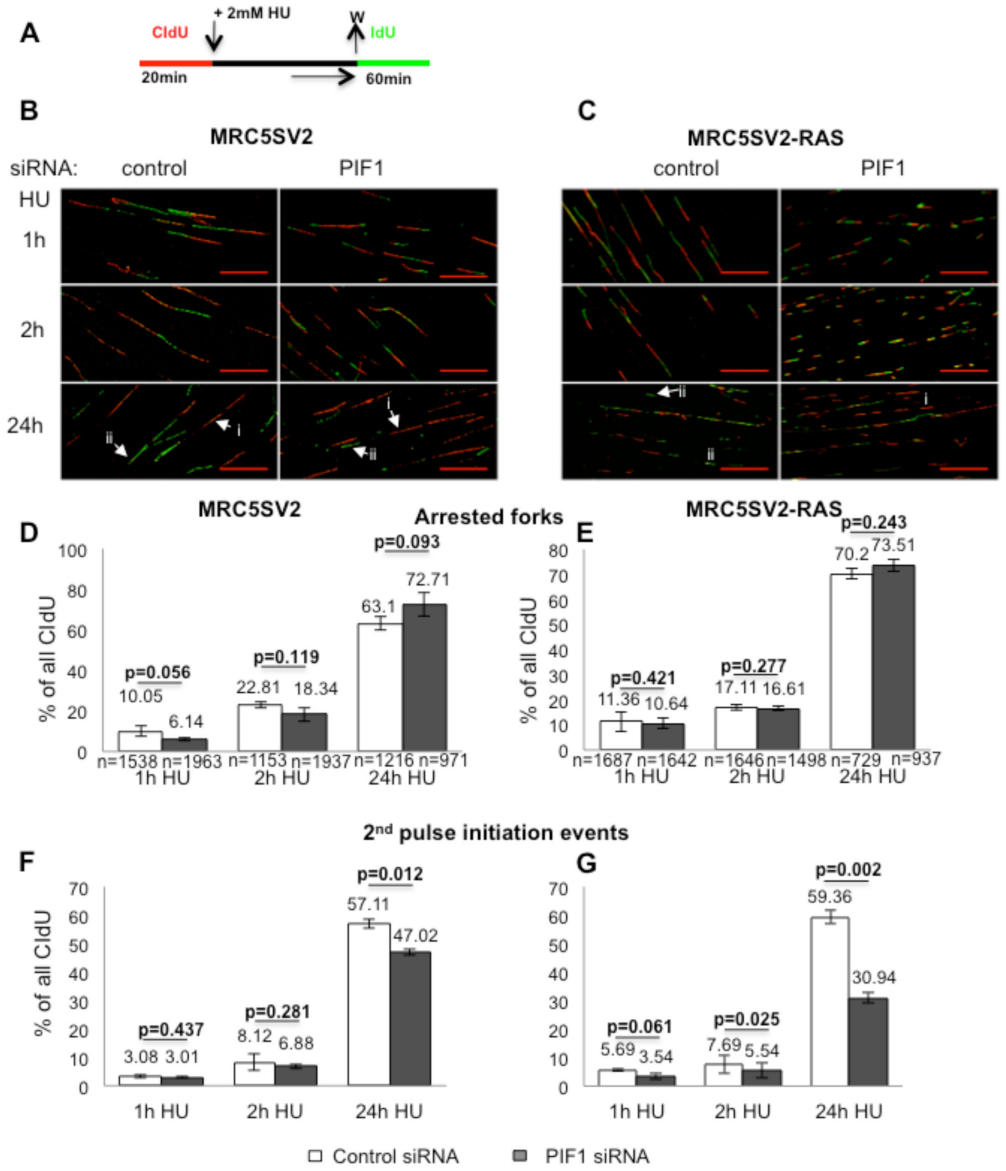
Effects of PIF1-depletion on recovery of stalled replication forks (**A)** Labelling protocol and, **(B, C)** Representative images (scale bars=20μm) of replication structures from parental and MRC5SV2-RAS cells (clone 2) treated with control or PIF1 siRNAs. White arrows marked with **(i)** or **(ii)** indicate arrested forks and new initiation events, respectively, **(D, E)** Bar graphs of arrested forks/termination events after indicated HU treatments, measured as in Figure 4, **(F, G)** New initiation events following the above treatments, assessed as second label initiations (only green) relevant to all CIdU tracks, respectively. The mean and SD of three independent experiments are shown, as well as *p* values of Student's *t* test. In Panels **D**, **E**, (n) represents the total number of CIdU-labelled replication structures (red, red-green and green-red-green) pooled from three independent repeats, while in Panels **F**, **G**, (n) is the same as in **D** and **E**, respectively.

Replication stress in oncogene-expressing cells depends on DNA precursor deficiency [40], mimicking cellular response after treatment with dNTP synthesis inhibitors. Based on the hypersensitivity of PIF1-depleted tumour cells [26] and RAS transformed fibroblasts to such inhibitors, we next investigated the potential role of PIF1 on recovery of DNA replication. In parental and RAS-transformed fibroblasts we assessed resumption of DNA synthesis after HU-induced arrest, using a reported assay [41] (Fig. 7A-C): Replication recovery, at individual forks, was assessed by the percentage of arrest/termination events as well as the percentage of new origins that fired during the second pulse. In particular, we measured only unilabelled tracks (only red/arrested forks and/or only green/new initiation events) which had at least 6μm distance between them, as previously defined [41]. This type of analysis avoids artifacts due to very slow rate of DNA polymerisation during long HU treatments [42], which could separate the two labelled tracks of a restarting replication fork. We found that upon control or PIF1 siRNA transfection followed by 1h HU treatment, the percentage of arrested forks in MRC5SV2 cells was 10.05% vs 6.14% (p=0.056), respectively, while in RAS-transformed MRC5SV2 was 11.36% vs 10.64% (p=0.421), respectively (Fig. 7D, E). The number of arrested forks was increased after 2h HU treatment but was not significant different between control and PIF1 siRNA treated cells, in both lines [22.81% vs 18.34% (p=0.119) and 17.11% vs 16.61% (p=0.277) in parental and RAS-transformed fibroblasts, respectively].

Taking together, the above data showed that after short or prolonged periods of S-phase arrest, the number of irreversibly stalled forks after inhibitor removal was not significantly affected by PIF1-depletion in either of the two cell lines, indicating that replication forks remained competent for DNA synthesis at control levels. In contrast, the firing of new origins upon PIF1-depletion was significantly reduced after 24h HU arrest in parental cells [% percentage of IdU pulse initiation events: 57.11 vs 47.02 (p=0.012) in control and PIF1 siRNA-treated cells, respectively (Fig. 7F)] and after 2 and 24h HU arrest in RAS-transformed fibroblasts. In particular, following control or PIF1 siRNA treatments, the % percentage of new origin firing in MRC5SV2-RAS cells during IdU pulse was 7.69 vs 5.54 (p=0.025) after 2h HU and 59.36 vs 30.94 (p=0.002) after 24h HU, respectively (Fig. 7G).

**Figure 8:**
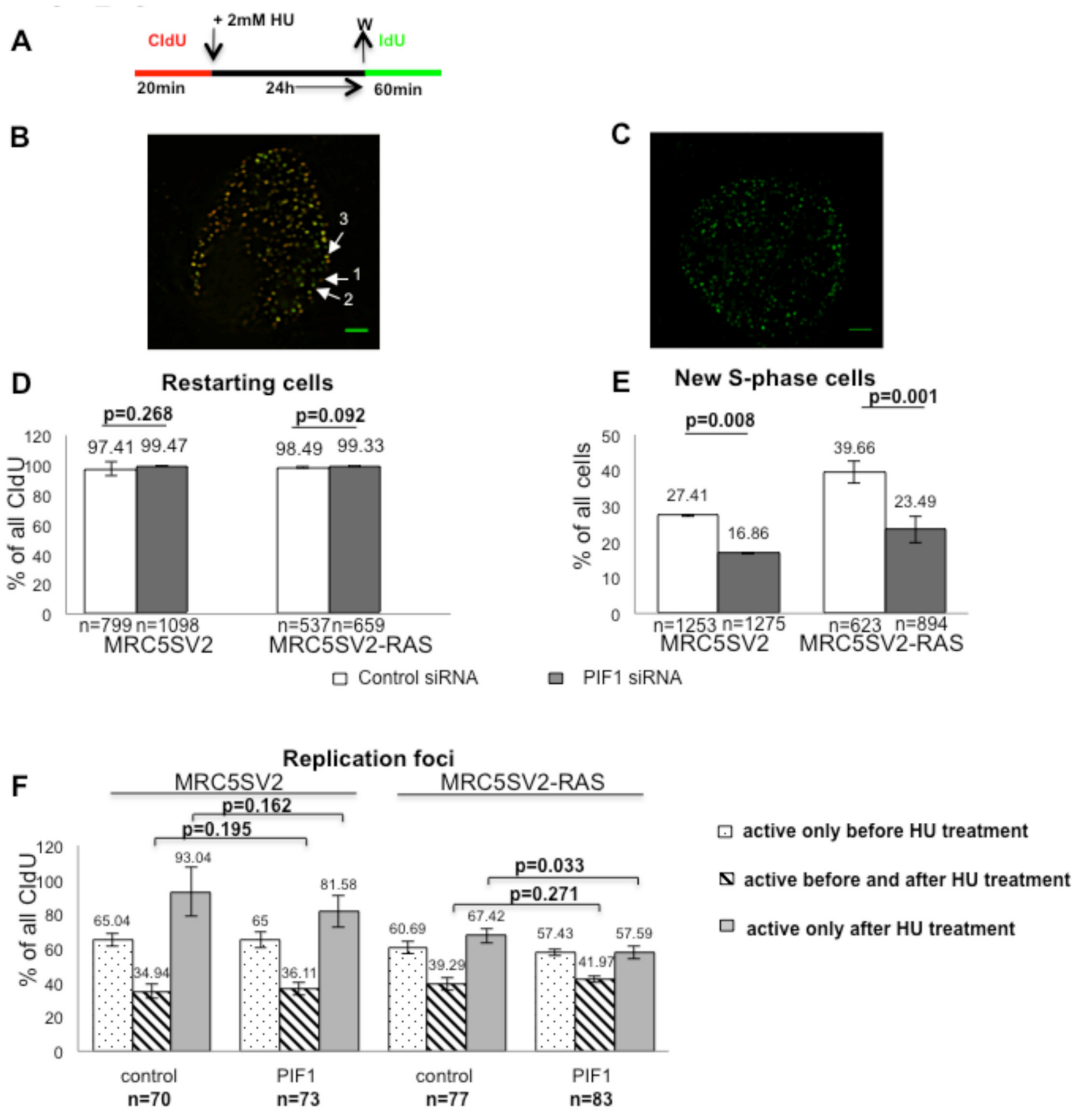
PIF1 is required for efficient resumption of DNA synthesis after prolonged replication arrest Cells were labelled and treated with HU as indicated in (**A)** and replication foci were detected in whole nuclei after pre-extraction, fixation and immunostaining for CIdU (red) and IdU (green). Single z-sections through representative nuclei of RAS-transformed cells (clone 2) resuming DNA synthesis under the above conditions are presented in (**B, C)** (Scale bars=2.5μm). The three-dimensional image stack for the cell in (**B)** is shown in Supplementary Information, video S1. Cells restarting replication are labelled with both CIdU and IdU and their percentages relative to all CIdU-labelled cells are presented in **(D)**. New S- phase cells are labelled only with IdU and are shown as percentages of all cells in **(E)**. **(F)** Bar graphs show the percentage of active replication foci during the different pulses normalised to total CIdU labelled foci in each nuclei. Nuclear foci representing replication sites that were active only during the first label (red) are marked with **(1)** in **(B)**, while those that have been activated during the second label (green) are marked with **(2).** Nuclear foci where red and green signals co-localized (yellow), represent replication sites that fired during the first pulse and remained active during the second, after HU arrest, are marked with **(3)**. The mean and SD (**D, E**) or SEM (**F**) of three independent experiments are shown, as well as *p* values of Student's *t* test. In **D** and **E**, (n) represents the total number of CIdU-labelled (red and red-green) or total cells (DAPI-stained), respectively, while in **F**, (n) represent the total number of single nuclei pooled from three independent repeats.

The effects of PIF1-depletion on replication recovery after prolonged S-phase arrest were also tested by immunofluorescence analysis of parental and two different clones of RAS-transformed fibroblasts pulse-labelled as above (Fig. 8A-C and Supplementary Information Fig. S5A-C). We found that the proportion of S-phase cells restarting replication (labelled with both CIdU and IdU), was not affected by PIF1-knockdown in either of the two cell lines [% percentage of restarting cells following control or PIF1 siRNA treatment: 97.41 vs 99.47 (p=0.268) and 98.49 vs 99.33 (p=0.092) in MRC5SV2 and MRC5SV2-RAS cells, respectively, Fig. 8D). Importantly, PIF1-knockdown significantly diminished the number of new cells entering S-phase (labelled only with IdU) in both parental and RAS-transformed lines (Fig. 8E). In particular, following PIF1-depletion the percentage reduction of new S-phase cells, relative to control cells, was 38.49% (p=0.008) in MRC5SV2 and 40.77% (p=0.001) in MRC5SV2-RAS cells (clone 2). Similar results were also obtained with a second clone (clone 3) of RAS-transformed fibroblasts [% percentage reduction of new S-phase cells: 36.32 (p=0.015) and 51.63 (p=0.0001) after treatment with PIF1 siRNA1 and PIF1 siRNA2, respectively, (Supplementary Information Fig. S5C)].

To examine further how PIF1-depletion affects new origin firing in S-phase cells restarting replication, we measured active DNA replication foci in single nuclei (Fig. 8F and Supplementary Information, video 1). As previously defined [43], we considered fired sites of replication before HU treatment that remained active and after inhibitor removal, the nuclear foci where signals from the CIdU (red), and the IdU (green) pulses were co-localized (yellow). New replication sites, activated after HU arrest, were assessed in each nucleus as the number of foci containing only IdU signal normalised to the total number of foci containing CIdU signal. We found that the percentage of fired replication foci that remained active after HU arrest was not affected by PIF1-depletion in either of the two cell lines [34.94% vs 36.11% (p=0.195) in MRC5SV2 and 39.29% vs 41.97% (p=0.271) in RAS-transformed MRC5SV2 cells upon control or PIF1 siRNA treatments, respectively]. In contrast, the percentages of new replication foci that fired after HU removal were slightly but significantly reduced upon PIF1-depletion only in RAS-transformed cells [67.42% vs 57.59% (p=0.033) in control or PIF1 siRNA-treated cells, respectively], while parental fibroblasts were not significantly affected [93.04% vs 81.58% (p=0.162) in control or PIF1 siRNA-treated cells, respectively].

Taken together, our data suggest a role for PIF1 in recovery of DNA replication. This role can facilitate the ability of the cells to resume prolonged-disrupted DNA synthesis by affecting firing of new origins and mainly by governing the number of new cells entering S-phase.

## DISCUSSION

Taken together, we have established a functional role for human PIF1 helicase in maintenance of unperturbed genome-wide DNA replication fork progression. This is the first time that a non-processive DNA helicase is reported to support processive DNA synthesis under normal cycling conditions in humans. Depletion of PIF1 resulted in slower replication fork rates and increased fork stalling under normal cycling conditions as well as after ligand-induced G4-structure stabilization. During replication, fork movement can be disrupted by folding of G4-structures at ssDNA GC-rich regions of lagging or leading strands [44–46]. In accord with PIF1's protective role against accumulation of stalled forks during treatments with DNA G4-ligands and evidence for nuclear co-localisation of PIF1 with binding regions of another G4-ligand [47], we speculate that PIF1 facilitates fork progression by directly binding and unwinding these secondary structures. This idea is also supported by the fact that the percentage reduction in fork rate of the whole genome upon PIF1-depletion was further increased in the presence of DNA G4-ligands, as we showed here. Upon PIF1-depletion, unresolved G4-structures can stall the DNA polymerase complex slowing or arresting replication forks and threatening genome stability. Increased replication stress and unscheduled initiation during oncogene overexpression [28] could affect G4-structure formation increasing the PIF1-dependence of fork progression, as we detected in RAS-transformed cells relative to parental cells. The proposed role of human PIF1 is in agreement with the reported role of the yeast ScPif1 in replication and stability of tandem arrays containing sequences with the potential to form G4-structures [48], as well as the highly conserved function of the Pif1 family helicases on suppression of DNA damage at G4-structures in the yeast genome [9]. However, it remains to be proved whether the effect of PIF1 knockdown on DNA replication progression is restricted to G4-regions of the human genome.

Despite the impairment of replication fork movement upon PIF1-depletion, cell cycle distributions of non-cancerous cells were almost normal, with a slight delay in S-phase, and growth and proliferation were mainly unaffected. This could be explained by an efficient re-priming of replication downstream of paused site [49, 50]. By this rationale, the observed increased fork stalling upon PIF1-depletion could be considered as temporal. This is also supported by the fact that DSB formation was not increased upon PIF1-depletion in either parental or RAS-transformed cells. By contrast, depletion of PIF1 becomes important for DNA replication and proliferation under conditions of oncogene-induced replication stress: in RAS-transformed lines, fork slowing was exacerbated leading to accumulation of cells in S-phase. Moreover, insufficient replication was accompanied by reduced proliferation rate, which can be explained by eventual growth arrest rather than an increase in spontaneous apoptosis.

When DNA synthesis is disrupted or damaged, restart of DNA polymerization at paused replication forks is essential for maintenance of genomic stability [51] and depends critically on proteins that stabilize forks, such as the BLM [52], WRN [53] and FANCJ/BACH1[54] helicases, and/or proteins required for homologous recombination, such as the RAD51[55, 56]. By contrast to the reported role of the above helicases on fork stabilization, PIF1 was dispensable for fork restart after replication disruption by HU treatment. However, when replication forks stall irreversibly, after prolonged HU arrest, resumption of replication occurs mainly through firing of new origins [41, 57]. We show here that PIF1 is required for replication recovery through its role in S-phase entry and origin firing at new replication sites. This function of PIF1 is redundant in normal growth conditions or after short periods of S-phase arrest. However, it could become critical when there is increased demand for origin firing from new replication sites, especially during oncogene-induced replication stress [58]. Recently, a consensus G4-motif, that can form G4-structure, has been reported to be associated with replication origin selection, usage efficiency and timing in human cells [59]. Furthermore, the above signature-motif was found to coincide with the specific RNA/ssDNA binding sequence of the human Origin Recognition Complex [60]. PIF1 could influence origin-firing processes through its ability to resolve such G4-structures [46]. In agreement with this mounting evidence, our data showed that in parental fibroblast upon PIF1-depletion the percentage reduction in fork rate of non-telomeric regions (35.13%) is similar to the percentage reduction in new S-phase entry (38.49%) suggesting that they may be correlated. This proposed function of PIF1 could also facilitate the recruitment of replication initiation factors to these structured sites. In support of this, reciprocal immunoprecipitation of C-terminal-FLAG-tagged PIF1 and endogenous CDC45 in human cancer cells has been reported [61], suggesting that PIF1 associates with the essential replication initiation co-factor CDC45 *in vivo* [62]. On the other hand, we cannot exclude an indirect role of PIF1 on resumption of DNA replication and S-phase entry. We have previously reported an increase in p21 protein levels upon PIF1 depletion in HCT116 cells [26]. However, this increase was detected only in a fraction of cells at the G1/S border that were not incorporating BrdU during resumption of replication after 24h arrest. Thus, it seems unlikely that the observed reduction of the active replication sites in RAS transformed cells restarting DNA synthesis upon PIF1 depletion is subject to p21 regulation although this may affect cells entering S-phase. Further studies will define the role of PIF1 on firing of replication origins and S-phase entry and whether this is restricted to sites with G4-structures.

Collectively, we report a critical role for PIF1 in the regulation of DNA replication under excessive replication stress. It would be interesting in the future to investigate whether the dependence of DNA replication on PIF1 is increased upon expression of oncogenes other than RAS. Moreover, our results of sensitisation of RAS transformed cells to gemcitabine upon PIF1 depletion warrant further studies of the effects of manipulation of PIF1 alone, as well as in combination with replication inhibitors or G4-ligands, on the survival of oncogene-driven tumours in model organisms.

## METHODS

### Cell lines and cultures

HCT116 cell line was obtained from American Type Culture Collection (Manassas, VA, USA). Human fibroblast cell line MRC5SV2, a derivative of MRC5 after transformation with the virus SV40, was obtained from the European Collection of Cell Cultures (Porton Down, Salisbury, UK). Cell line identity was confirmed by DNA fingerprinting (LGC Standards, Teddington, UK), and mycoplasma-free status was verified using the Mycoplasma PCR ELISA kit (Roche-Applied Biosciences, Mannheim, Germany).

HCT116 strains conditionally over-expressing wild type or mutant PIF1 proteins were obtained by using the Flp-In T-REX system (Invitrogen, Carlsbad, CA, USA). Briefly, an HCT116 Flp-In T-REX host cell line was generated according to manufacturer's instructions. Full length of the hPif1a coding region, carrying or not the E307Q substitution and having or not a FLAG-tag at the C-terminus, were cloned into the pcDNA5/FRT/TO vector and integrated into the HT-REX cell line via Flp recombinase-mediated DNA recombination at the FRT site. Tetracyclin inducible stable lines were isolated by selection for blasticidin and hygromycin resistance.

MRC5SV2 strains stably over-expressing the mutant HRAS^G12V^ were generated by transfection of HRAS^G12V^ cDNA (Addgene plasmid 22252) after subcloning into pCAG-Flox vector. Stable isolates expressing HRAS^G12V^(clones 2 and 3) were obtained by selection in puromycin.

MRC5SV2 and HRAS^G12V^-MRC5SV2 cells used in all the experiments were derived from cultures of no more than 15 passages and were negative for senescence-associated *β*-galactosidase staining (9860; Cell Signaling, Danvers, MA, USA).

Cells were maintained in DMEM supplemented with 10% fetal bovine serum (FBS). HU, SYUIQ-5 and N-(3-Aminopropyl) piperidine (Sigma-Aldrich, St Louis, Missouri, USA) were used at a concentration 2mM, 1μM and 5μM, respectively. Cells were irradiated using a CIS IBL 437 Cs-137 irradiator (CNRS, Gif-Sur-Yvette, France).

### SiRNA transfection

For HRAS depletion a pool of four siRNA duplexes [LQ-004142-00, Dharmacon (Lafayette, CO, USA)] with the following sense sequences were used:

GAACCCUCCUGAUGAGAGU, AGACGUGCC UGUUGGACAU, GGAAGCAGGUGGUCAUUGA and GAGGAUGCCUUCUACACGU. PIF1 siRNAs with the sense sequences GGCCAGAGCAUCUUCUUCATT (siRNA 1) and CCCUUCAGAGCCUAACCAATT (siRNA 2) were obtained from Applied Biosystems (Ambion, Carlsbad, CA, USA). Negative Control siRNAs with no sequence homology in the human genome, were obtained from Eurogentec (OR-0030-NEG, Seraing, Liege, Belgium). Lipofectamine 2000 (Invitrogen) was used to transfect siRNA duplexes into cells, according to manufacturer's instructions. The cells were incubated for 48 hours before further treatment.

### Flow cytometry and Pulse-Field Gel Electrophoresis

Flow cytometry and Pulse-Field Gel Electrophoresis have described previously [63]. Further data analysis was performed using the FlowJo software (http://www.flowjo.com).

### Protein extraction and Western Blotting

Whole-cell extracts were prepared, fractionated, and blotted onto nitrocellulose (Whatman Schleicher and Schuell, Dassel, Germany) as described previously [64]. Proteins were detected with the ECL system (GE Healthcare, Little Chalfont, UK) and visualized with the Fujifilm LAS-3000 imager (Fujifilm, Tokyo, Japan). The following specific antibodies were used: ATM (sc- 23921 Santa Cruz, Dallas, Texas, USA), γH2AX (2577; Cell Signaling), CHK1 (2360; Cell Signaling), β-actin (A-5060; Sigma-Aldrich), RPA2 (NA19L; Calbiochem, Billerica, MA, USA), phospho-ATM (Ser1981) (2152-1; Epitomics, Burlingame, CA, USA), phospho-CHK1 (Ser345) (2349; Cell Signalling), HRAS (OP38; Calbiochem).

### RNA purification and qPCR analysis

Extraction and purification of total RNA, DNase I treatment and first-strand cDNA synthesis have described previously [26]. qPCR reactions were run on a 7900HT Fast Real-Time PCR system (Applied Biosystems) with SYBR Green reagents (Quantace, London, UK) and 0.25 μM of each primer. The following PCR conditions were used: Heating for 10 min at 95°C followed by amplification for 45 cycles (15sec 95°C / 15sec 59°C / 25sec 72°C). All samples were analysed in duplicate. SDS 2.2.1 software (Applied Biosystems) was used for data analysis and evaluation. Primer3plus (http://www.bioinformatics.nl/cgi-bin/primer3plus/primer3plus.cgi) and NCBI (http://www.ncbi.nlm.nih.gov/tools/primer-blast) programs were used to design the following primers:

PIF1 (i): 5′ccctggattgtgtggagatt 3′ (Forward);
PIF1 (ii): 5′actccagactgaggctcctg 3′ (Reverse);
PIF1 (iii): 5′cctatgtggccctttctcg 3′ (Forward);
PIF1 (iv): 5′ggtttgggtccatgttctcc 3′ (Reverse);
ATPS6: 5′ccttatgagcgggcacagt 3′ (Forward);
ATPS6: 5′ ccagggctattggttgaatg 3′ (Reverse);
Primers for the U1 snRNA were from Hautbergue, G. [65]

### DNA-fibre analysis

We have used pulse-labeling conditions that support DNA polymerization at normal rates according to [31]. Labelled-cells were harvested, lysed and DNA spreading, and immunostaining were performed as previously described [58]. For CIdU detection, a rat anti- Bromodeoxyuridine (BrdU, clone BU1/75(ICR1); AbD Serotec) were used in a dilution 1:500 and detected with an Alexa-555 conjugated goat anti-rat IgG (A-21434; Molecular Probes, Eugene, Oregon, USA). For IdU detection, a mouse anti-BrdU [(clone B44); BD, Franklin Lakes, NJ, USA] were used in a dilution 1:500 and detected with an Alexa-488 conjugated F(ab')_2_ fragment of goat anti-mouse IgG (A-11017; Molecular Probes). Antibody dilutions and washes were performed in PBS containing 1% BSA and 0.1%Tween 20. Coverslips were mounted in Fluoroshield medium (F6182; Sigma-Aldrich).

### Immunodetection of replication sites in whole nuclei

Cells were grown on glass coverslips, treated with siRNAs and pulse-labelled as described above. Cells were pre-extracted with a buffer containing 10mM HEPES pH 7.9, 10mM KCI, 1.5mM MgCI_2_, 0.34M sucrose, 10% glycerol, 0.1% Triton X-100 and 1mM PMSF for 5min at 4°C, fixed with 3% paraformaldehyde, 2% sucrose in PBS for 10min at R/T and permeabilized with 0.5% Triton X-100 in PBS for 5min at 4°C. DNA on coverslips was denaturated with 2N HCI for 40min at R/T, followed by neutralization with PBS buffer. CIdU and IdU signals were detected with the antibodies described above. Coverslips were mounted in Vectashield medium with 4′,6-Diamidino-2-phenylindole dihydrochloride [(DAPI), H-1500; Vector Laboratories Inc., Burlingame, CA, USA].

### Immunofluorescence analysis

Immunofluorescence at replication structures and whole nuclei was visualized using an Olympus FV1000 confocal BX61 upright microscope equipped with X 60(1.42 NA) or X 100 (1.4 NA) objective lens, respectively. Images were captured and analyzed by Fluoview 3.1 software (Olympus, Shinjuku, Tokyo, Japan). Measurements of labelled tracks were performed in micrometres, by using the ImageJ software (http://rsbweb.nih.gov/ij/), and converted to kilobases with the reported(30) factor 1μm=2.59Kb.

For co-localisation experiments, serial optical sections of 0.2μm were collected through nuclei using a wide-field fluorescence microscope (DeltaVision DV3; Applied Precision, Issaquah, WA, USA) equipped with a 100X (1.4 NA) objective (Olympus). SoftWorx (Applied Precision) software was used to deconvolute image stacks, which were further analysed with Volocity (Perkin Elmer, Waltham, MA, USA) software. Nuclear foci with size >0.05 and <1.0μm, labelled with CIdU and/or IdU were measured in each cell.

### MTT proliferation assay

Cells seeded at a density of 1,000 cells/well of a microplate were transfected the next day with indicated siRNAs and left to grow for another 3–5 days. At that point, MTT [3-(4,5-dimethylthiazol-2-yl)-2,5-diphenyltetrazolium bromide] reagent (11465007001, Roche- Applied Biosciences) was added at a final concentration of 1mg/ml followed by incubation at 37°C for 3hrs. Solubilization of the produced formazan was achieved by sodium dodecyl sulphate (SDS) and further incubation at 37°C O/N. Concentration of the soluble product was determined by optical density at 540nm, using standard microplate absorbance reader.

### Statistical analysis

Statistical analysis of the data was performed using Student's *t*-test from paired samples. P value was considered statistically significant when was <0.05. Error bars in bar graphs represent the standard deviation in the values (SD) or the standard error of the mean (SEM) of at least three independent experiments, as stated in Figure legends. The exact numbers (n) are listed as well.

## SUPPLEMENTARY INFORMATION TEXT


