# Supplementary material for: Human PIF1 helicase supports DNA replication and cell growth under oncogenic-stress
Source: Oncotarget. 2014 Nov 5;5(22):11381–98. doi: 10.18632/oncotarget.2501 (PMC4294361; doi:10.18632/oncotarget.2501)
Supplement: Supplementary file 1 [file oncotarget-05-11381-s001.pdf]

SUPPLEMENTARY INFORMATION TEXT

Depletion of PIF1 protein in human cells.

An important consideration in interpreting our data is whether siRNA treatments affect all the isoforms of PIF1 protein, nuclear and mitochondrial. To address this, we quantitated expression levels of the major transcript hPif1a, the expression of which gives rise to a nuclear 70kDa polypeptide, by using qPCR, relative to the levels of total Pif1 mRNA in our cell lines. We found that the hPif1a transcript level constituted the vast majority of the total Pif1 mRNA in both parental and RAS-transformed MRC5SV2 (96% and 98%, respectively; Supplementary Information Fig. S1A-C).

Furthermore, immunofluorescence analysis in a HCT116 cell line, conditionally expressing a recombinant C-terminal FLAG-tagged of the hPif1a cDNA (HTREX-wtPIF1-CFLAG line, described in **Methods**), using a FLAG antibody, showed marked enrichment of the signal in the nucleus relative to the cytoplasm (Supplementary Information Fig. S1D). Similarly, Western Blot analysis of total protein extracts from the above cell line, by using the same FLAG antibody, detected only one specific band at size approximately 72kDa, that corresponds to the nuclear FLAG-tagged PIF1 isoform (Supplementary Information Fig. S1E). An antibody raised against the human PIF1 protein [26] recognized two specific bands, a main one at 72kDa and a smaller one, a bit below 50kDa. The last band could correspond to the short mitochondrial PIF1 isoform. However, it is not detected with the FLAG antibody. Thus, we tend to believe that this short band is a degradation product of the main band of 72kDa.

Taken together, we conclude that the PIF1-siRNA treatments in our experiments primarily produce knockdown of the nuclear PIF1 because the presence of other human PIF1 isoform(s) is almost at undetectable levels in our experimental system.

SUPPLEMENTARY FIGURES AND TABLE

Supplementary Table S1. Cell cycle distribution in parental and RAS transformed MRC5SV2 cells after single treatment with control or PIF1 siRNAs and co-treatments with control/HRAS and PIF1/HRAS siRNA duplexes for indicative time

	48h post transfection				72h post transfection			
siRNA	control		PIF1		control		PIF1	
MRC5SV2								
	Mean	SD	Mean	SD	Mean	SD	Mean	SD
SubG1	2.27	2.06	1.99	0.79	2.47	0.29	3.76	2.03
G1	52.29	3.73	53.77	3.24	53.81	2.67	48.81	4.36
S	21.24	3.14	24.32	2.09	21.07	5.96	28.69	4.96
G2/M	22.28	7.43	18.29	5.21	20.77	6.58	17.46	8.25
MRC5SV2-RAS cl2								
SubG1	3.14	1.53	4.48	1.62	7.09	1.75	11.86	2.49
G1	46.18	3.65	42.52	5.42	40.27	0.41	34.84	7.12
S	20.82	1.68	32.52	1.28	20.25	4.77	31.38	3.73
G2/M	26.72	3.93	18.43	2.69	28.33	1.93	17.31	7.51
MRC5SV2-RAS siRNAHRAS								
SubG1	2.22	0.45	5.59	1.76	4.35	1.4	7.87	2.87
G1	45.76	3.37	41.13	6.42	46.56	3.28	39.43	3.8
S	19.76	3.22	28.24	0.84	21.36	5.6	26.82	5.87
G2/M	27.11	4.78	22.09	7.29	24.16	5.87	22.03	7.15

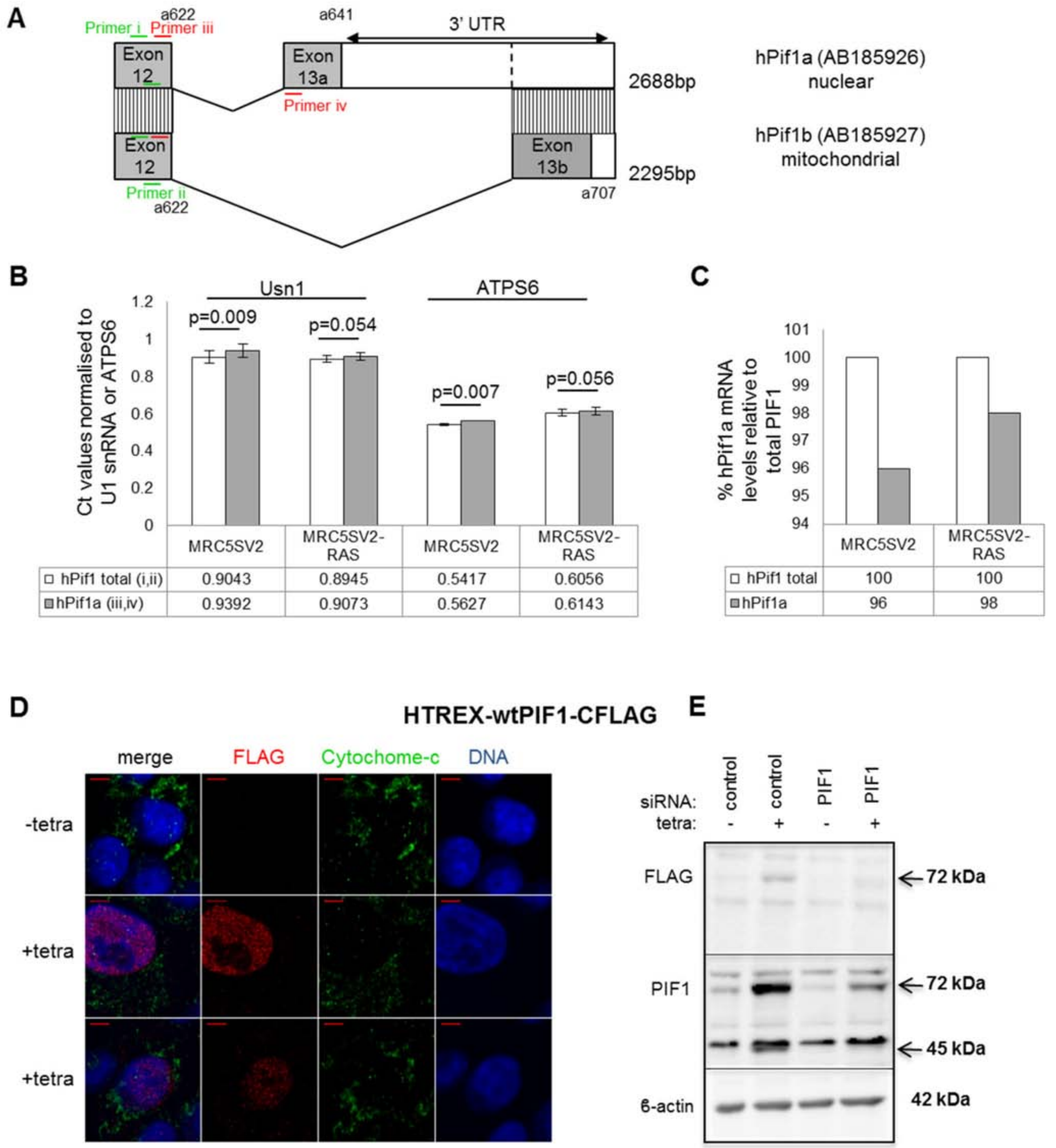

Supplementary Figure S1: Expression levels of human PIF1 isoforms in our experimental systems, (A) Schematic diagram with the C-terminal part of the two transcripts of the human PIF1 gene, as previously reported [19]. The sites of the two pairs of primers that have been used for amplification of total and hPif1a cDNAs are indicated with green and red lines, respectively, **(B)** Quantitative RT-PCR analysis of total and hPif1a mRNA, relative to U1snRNA and ATPS6, **(C)** Bar graphs with the % percentage of hPif1a mRNA relative to total PIF1 mRNA, extracted from panel **B**, **(D)** Immunofluorescence images (scale bars=5μm) of FLAG (red) and Cytochrome C (green) antibody staining in HTREX-wtPIF1-CFLAG cells, after induction with tetracyclin or not of the expression of a C-terminal FLAG-tagged wtPIF1. DNA was counterstained with DAPI (blue), **(E)** Western blot analysis in total protein extracts of HTREX-wtPIF1-CFLAG cells after induction or not with tetracyclin and treatment with the indicated siRNAs for 48h. Levels of β-actin were served as loading marker. Specific bands are indicated with arrows. Results in Panel **B** represent means of at least three independent experiments with standard deviations (SD) indicated by error bars as well as *p* values of Student's *t* test.

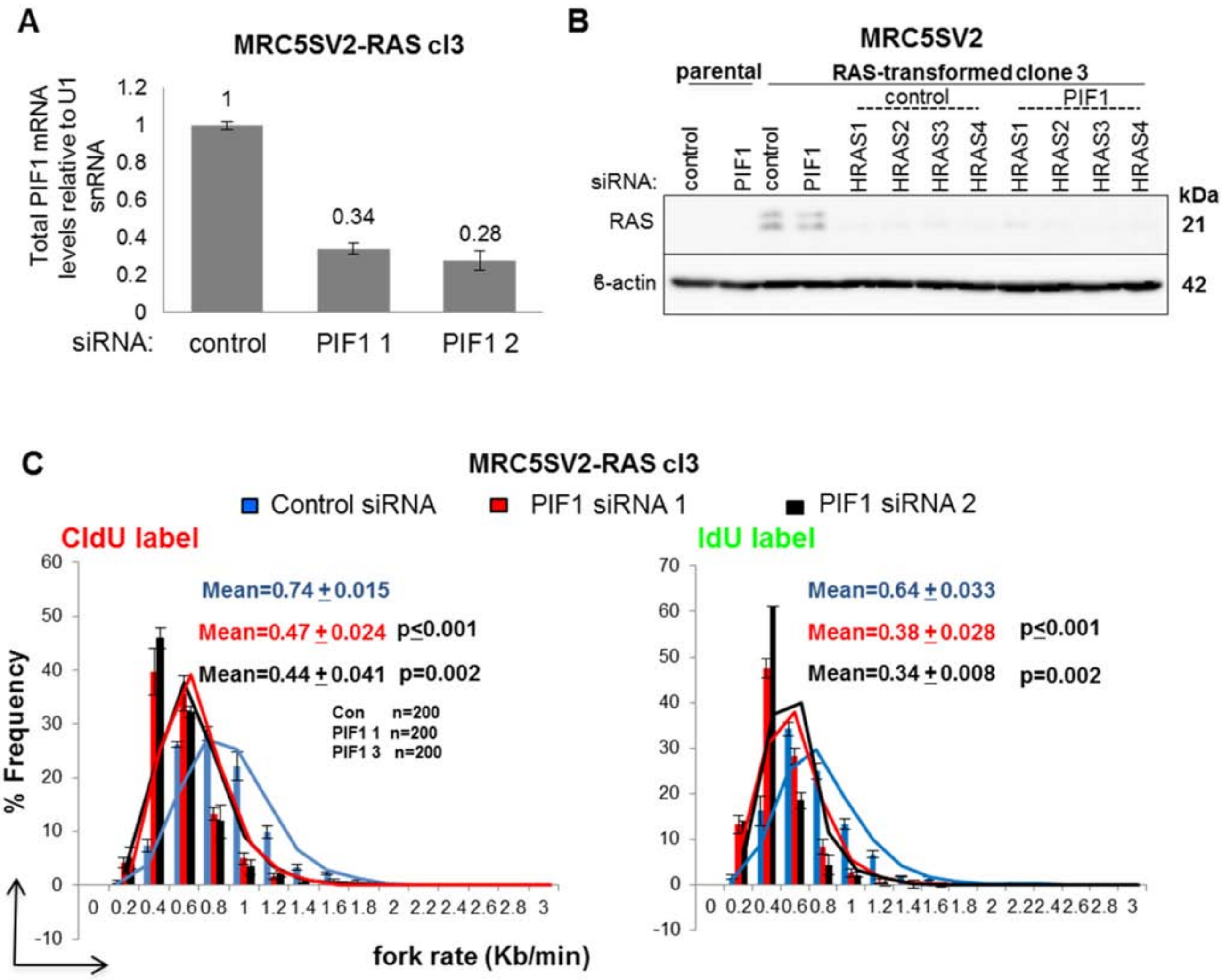

Supplementary Figure S2: Effects of PIF1 depletion on replication fork movement of RAS transformed MRC5SV2 cells (clone 3). **(A)** Quantitative RT-PCR analysis of total PIF1mRNA and, **(B)** Western blot analysis of RAS expression in H-RAS^{G12V}-transformed MRC5SV2 cells (clone 3) after indicated siRNA treatments. When is not indicated, a pool of the two siRNAs, PIF1 1 and PIF1 2 was used for the depletion of this protein. β-actin levels served as loading controls, **(C)** Cells were labeled and fork rates were measured as described in Figure 1. Distribution of fork rates during CIdU and IdU pulses is shown in left and right panels, respectively. Data bars present the mean of three independent experiments and error bars represent SD in **A** and SEM in panels **C**. In fork rate distributions, the total mean and SD of the three repeats are also shown, as well as *p* values of Student's *t* test. The exact numbers (n) for each experiment are listed as well.

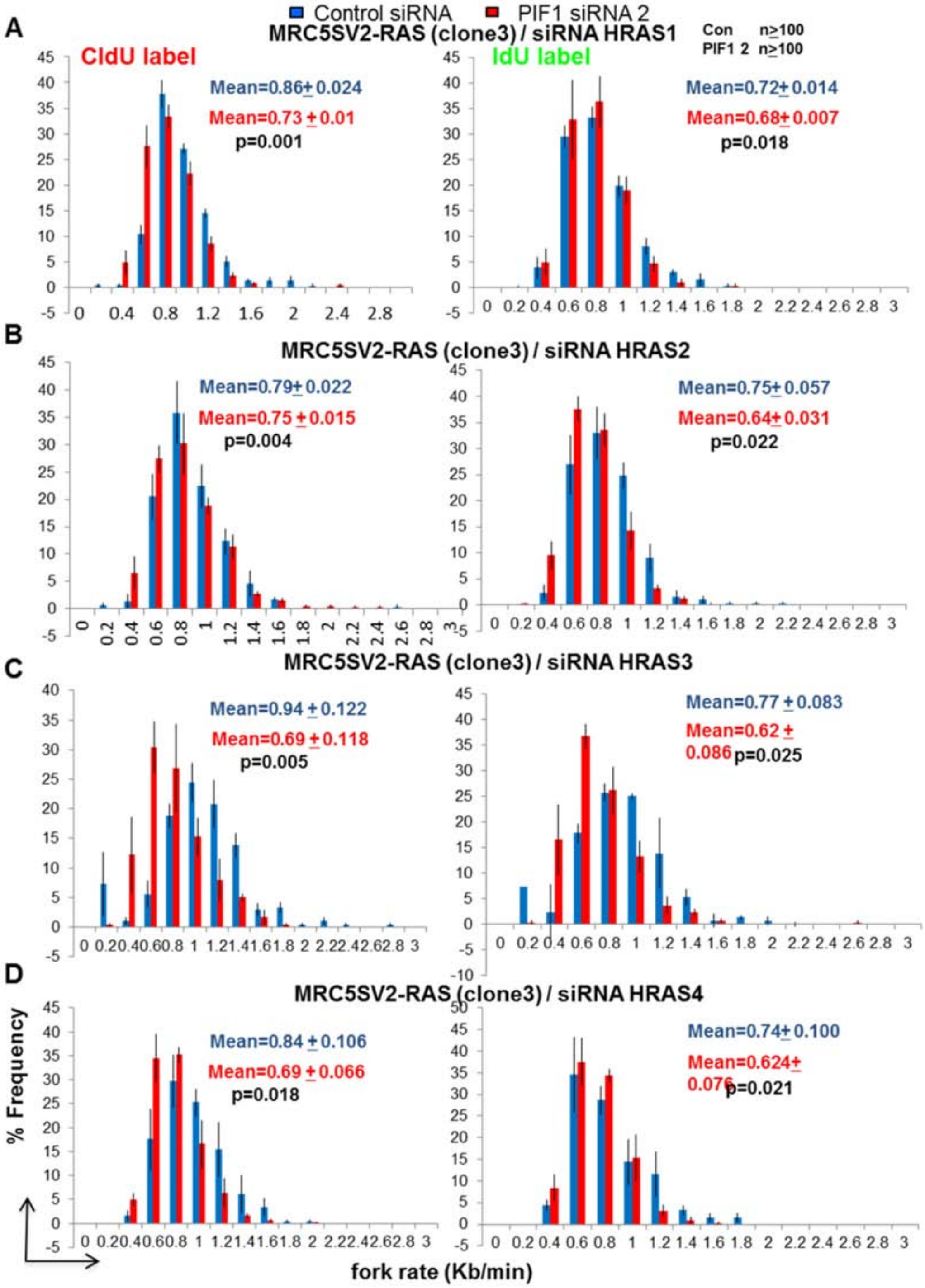

Supplementary Figure S3: Depletion of HRAS diminishes the effects of PIF1 depletion in RAS transformed MRC5SV2 cells (clone 3). **A-D**. Cells were treated with the indicated siRNAs, labelled and fork rates were measured as in Figure 1. Fork rates distribution during CIdU and IdU pulses is shown in left and right panels, respectively. Data bars present the mean of three independent experiments and error bars represent SEM. The total mean and SD of the three repeats are also shown, as well as *p* values of Student's *t* test and numbers (n) for each experiment.

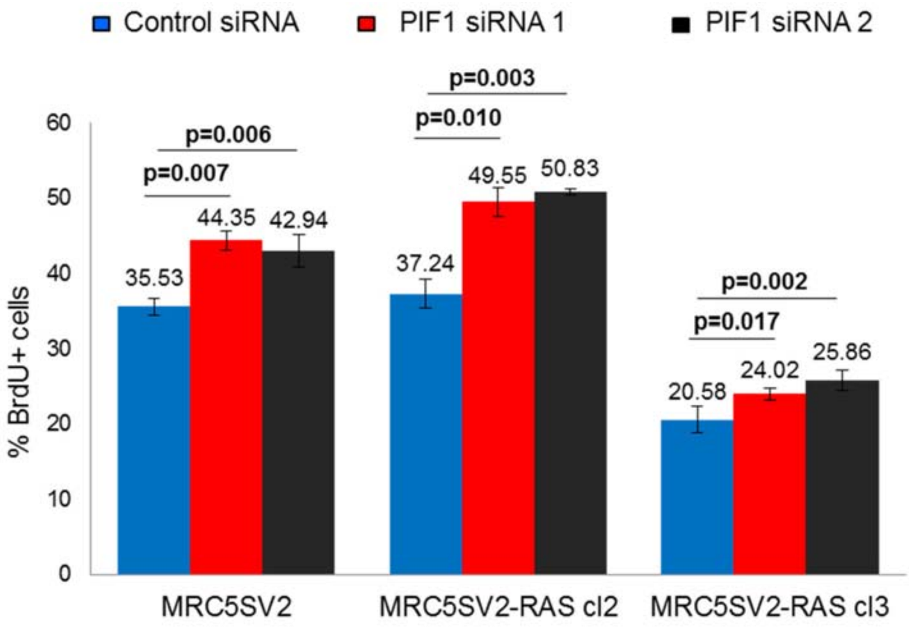

Supplementary Figure S4: BrdU incorporation in MRC5SV2 and RAS transformed MRC5SV2 cells (clones 2 and 3) under normal cycling conditions. Asynchronous cultures of cells treated with the indicated siRNAs were labelled with BrdU for 20min and then whole nuclei were pre-extracted, fixed and immunostained for BrdU. Immunofluorescence was detected with microscopy. Bar graphs show the number of cells positive for BrdU as percentages of all cells. The mean and SD of three independent experiments are shown, as well as *p* values of Student's *t* test. At least 300 cells were counted in each repeat for each condition.

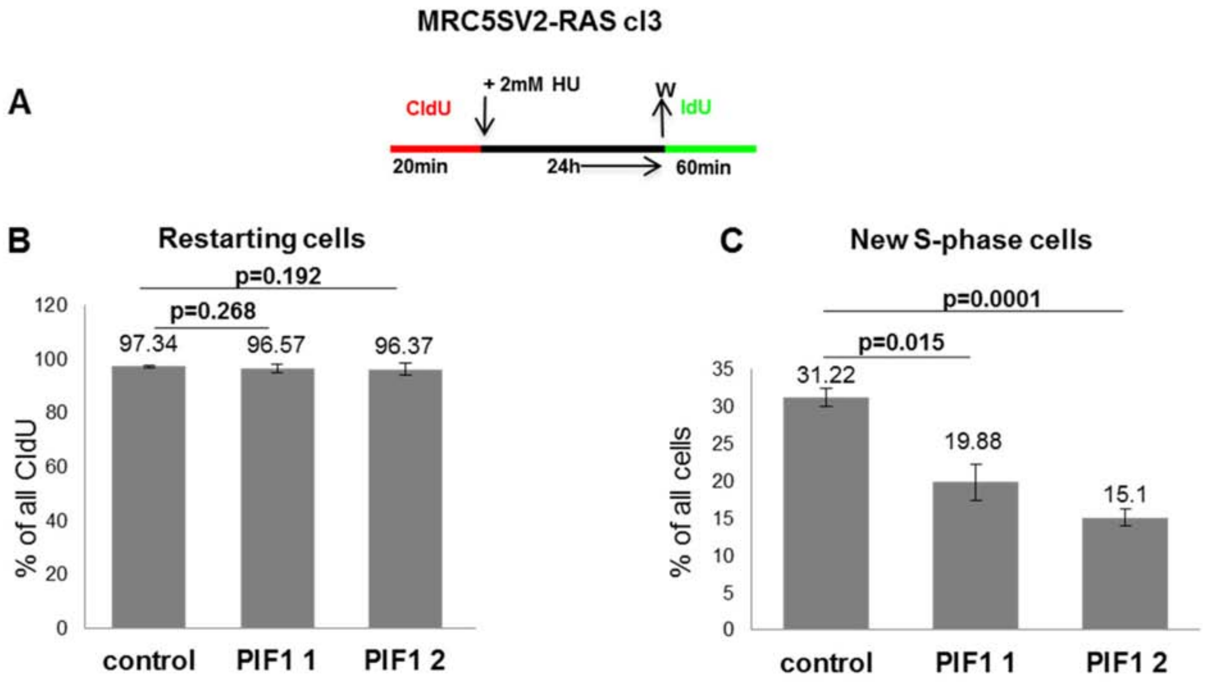

Supplementary Figure S5: Effects of PIF1 depletion in resumption of DNA replication after prolonged S-phase arrest in RAS transformed MRC5SV2 cells (clone 3). (A) Cells treated with the indicated siRNAs were pre-labelled with CIdU before arrest in S-phase with HU for 24h. When HU was removed, cells were left to recover with further IdU labelling. Restarting cells and new cells entering the S-phase were assessed as described in Figure 8. **(B)** Bar graph present the percentages of cells restarting replication relative to all CIdU-labelled cells, **(C)** New S- phase cells are shown as percentages of all cells. The mean and SD of three independent experiments are shown, as well as *p* values of Student's *t* test. At least 250 cells were counted in each repeat for each condition.

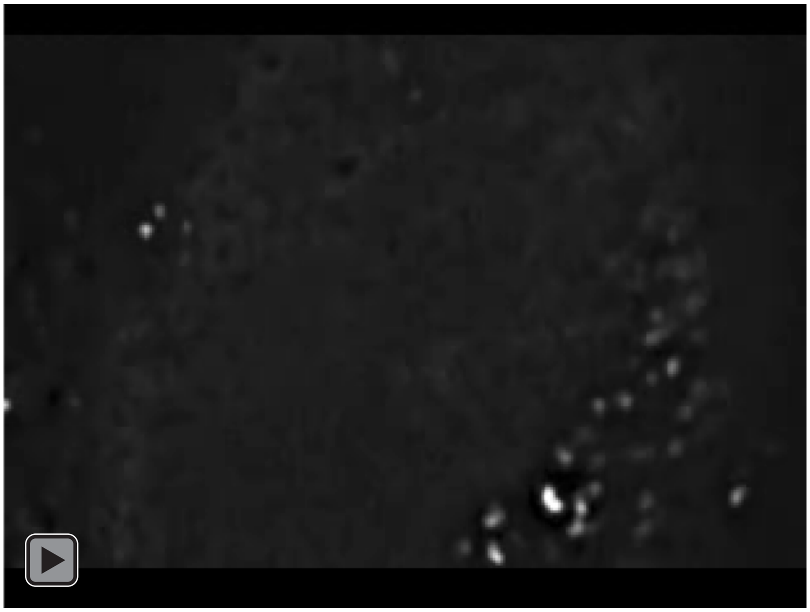

Supplementary Video S1
